# Supplementary material for: Increasing Prevalence of HIV-1 Transmitted Drug Resistance in Portugal: Implications for First Line Treatment Recommendations
Source: Viruses. 2020 Oct 30;12(11):1238. doi: 10.3390/v12111238 (PMC7693025; doi:10.3390/v12111238)
Supplement: Supplementary file 1 [file viruses-12-01238-s001.pdf]

Additional File 1

Title: Increasing prevalence of HIV-1 Transmitted Drug Resistance in Portugal: implications for first line treatment recommendations

Statistical Analysis

In the univariate analysis of factors associated with HIV drug resistance the following covariates were separately included: sex, age at diagnosis, risk groups, geographic region of origin, subtype, CD4 value and viral load. Factors significantly associated with TDR in a univariate analysis ($p<0.05$), factors with a p value less than 0.2[1] and factors considered with biologic value (sex and age at diagnosis) were included into multiple logistic regression.

Patterns of TDR, ADR and for the prevalence of mutations over time were calculated by logistic regression, using R 3.5.1. For TDR, ADR and mutations the logistic regression model was calculated for each class of resistance, with Odds Ratio (OR) and 95% confidence intervals (CI). Results were considered statistically significant when p-values were below 0.05.

[1] Harrel, F. E., 2010. Regression Modelling Strategies: with applications to linear models, logistic regression, and survival analysis, Springer series in statistic. Springer, New York.

Table S1- Trends of transmitted and acquired drug resistance between 2014 and 2017. NRTI, Nucleotide reverse transcriptase inhibitors; NNRTI, Non-nucleotide reverse transcriptase inhibitors; PI, Protease inhibitors; CI, confidence interval; OR, odds ratio.

**Years**	**Transmitted drug resistance (TDR)**	**OR (95% CI)**	**p for trend**
**2014-2017**	Any DRMs	1.169 (1.042-1.311)	0.008
	NRTI resistance	1.337(1.127-1.587)	0.001
	NNRTI resistance	1.132 (1.003-1.278)	0.044
	PI resistance	0.943 (0.760-1.170)	0.595
	Single class resistance	1.111 (0.972-1.269)	0.122
	Dual classes resistance	1.251 (1.003-1.561)	0.047
	PI + NRTI resistance	1.019 (0.560-1.855)	0.950
	PI + NNRTI resistance	1.511 (0.636-3.590)	0.350
	NRTI+NNRTI resistance	1.270 (0.992-1.626)	0.058
	Triple class resistance	1.361 (0.728-2.546)	0.334
**Years**	**Acquired drug resistance (ADR)**	**OR (95% CI)**	**p for trend**
**Sep 2014-2017**	Any DRMs	0.983 (0.839-1.153)	0.837
	NRTI resistance	0.987 (0.838-1.162)	0.874
	NNRTI resistance	0.985 (0.836-1.160)	0.852
	PI resistance	0.945 (0.729-1.225)	0.669
	Single class resistance	1.057 (0.869-1.286)	0.580
	Dual classes resistance	0.908 (0.756-1.089)	0.298
	PI + NRTI resistance	0.831 (0.532-1.296)	0.413
	PI + NNRTI resistance	1.754 (0.202-2.813)	0.674
	NRTI+NNRTI resistance	0.936 (0.772-1.135)	0.503
	Triple class resistance	1.122 (0.775-1.623)	0.542

Table S2- Proportion of transmitted drug resistance and of acquired drug resistance for single, double and triple classes and for associations of drug classes between 2001 and 2017. NRTI, Nucleotide reverse transcriptase inhibitors; NNRTI, Non-nucleotide reverse transcriptase inhibitors; PI, Protease inhibitors; CI, confidence interval; OR, odds ratio.

	ART-Naive patients						ART-Experienced patients					
	Single class (n; %)	Double Class (n; %)	Triple Class (n; %)	NRTI + NNRTI (n; %)	NRTI+PI (n; %)	NNRTI+PI (n; %)	Single class (n; %)	Double Class (n; %)	Triple Class (n; %)	NRTI+ NNRTI (n; %)	NRTI+PI (n; %)	NNRTI+PI (n; %)
2001	4 (8.9)	5 (11.1)	3 (6.7)	2 (4.4)	2 (4.4)	1 (2.2)	35 (44.6)	109 (48.7)	50 (22.3)	42 (18.8)	65 (29.0)	2 (0.9)
2002	4 (5.7)	2 (2.9)	0 (0.0)	0 (0.0)	2 (2.9)	0 (0.0)	70 (20.3)	155 (44.9)	78 (22.6)	57 (16.5)	97 (28.1)	1 (0.3)
2003	11 (4.4)	7 (2.8)	2 (0.8)	2 (0.8)	5 (2.0)	0 (0.0)	53 (18.8)	148 (52.4)	45 (16.0)	97 (34.4)	47 (16.7)	4 (1.4)
2004	7 (4.5)	3 (1.9)	0 (0.0)	1 (0.6)	2 (1.3)	0 (0.0)	83 (21.4)	208 (53.7)	41 (10.6)	143 (37.0)	62 (16.0)	3 (0.8)
2005	10 (7.6)	6 (4.5)	1 (0.8)	3 (2.3)	3 (2.3)	0 (0.0)	60 (18.9)	142 (44.9)	36 (11.4)	103 (32.7)	37 (11.7)	2 (0.6)
2006	9 (4.7)	1 (0.5)	0 (0.0)	0 (0.0)	0 (0.0)	1 (0.5)	63 (17.0)	175 (47.2)	40 (10.8)	135 (36.4)	40 (10.8)	0 (0.0)
2007	22 (5.2)	3 (0.7)	0 (0.0)	1 (0.2)	1 (0.2)	1 (0.2)	60 (20.2)	109 (36.7)	25 (8.4)	78 (26.3)	26 (8.8)	5 (1.7)
2008	44 (7.6)	9 (1.5)	2 (0.3)	7 (1.2)	0 (0.0)	2 (0.3)	51 (20.0)	72 (28.2)	11 (4.3)	56 (22.0)	15 (5.9)	1 (0.4)
2009	38 (6.1)	9 (1.5)	0 (0.0)	6 (1.0)	1 (0.2)	2 (0.3)	71 (23.4)	82 (27.1)	16 (5.3)	66 (21.8)	15 (5.0)	1 (0.3)
2010	51 (7.9)	4 (0.6)	1 (0.2)	2 (0.3)	0 (0.0)	2 (0.3)	62 (26.1)	61 (25.6)	6 (2.5)	50 (21.6)	11(4.6)	0 (0.0)
2011	44 (6.7)	8 (1.2)	0 (0.0)	2 (0.3)	4 (0.6)	2 (0.3)	38 (26.0)	36 (24.6)	6 (4.1)	28 (19.2)	7 (4.8)	1 (0.7)
2012	39 (9.2)	3 (0.7)	0 (0.0)	2 (0.5)	0 (0.0)	1 (0.2)	31 (29.5)	30 (28.6)	1 (1.0)	23 (21.9)	6 (5.7)	1 (1.0)
2013	53 (9.7)	7 (1.3)	0 (0.0)	5 (0.9)	1 (0.2)	1 (0.2)	22 (22.2)	26 (26.3)	3 (3.0)	21 (21.2)	4 (4.0)	1 (1.0)
2014	41 (6.9)	11 (1.8)	0 (0.0)	9 (1.5)	2 (0.3)	0 (0.0)	31 (20.9)	42 (28.4)	5 (3.4)	35 (23.6)	6 (4.0)	1 (0.7)

2015	44 (6.9)	16 (2.5)	2 (0.3)	13 (2.0)	2 (0.3)	1 (0.2)	23 (18.7)	30 (24.4)	9 (7.3)	25 (20.3)	5 (4.1)	0 (0.0)
2016	58 (8.1)	21 (2.9)	3 (0.4)	15 (2.1)	3 (0.4)	3 (0.4)	20 (18.9)	33 (31.1)	2 (1.9)	28 (26.4)	4 (3.8)	1 (0.9)
2017	56 (10.0)	23 (3.7)	3 (0.5)	20 (3.2)	2 (0.3)	1 (0.2)	25 (24.5)	20 (19.6)	7 (6.9)	18 (17.6)	2 (2.0)	0 (0.0)

Table S3- Time trends of selected mutations with prevalence greater than 0.1% for ART-NP and 1.0% for ART-TP. NRTI, Nucleotide reverse transcriptase inhibitors; NNRTI, Non-nucleotide reverse transcriptase inhibitors; PI, Protease inhibitors; CI, confidence interval; OR, odds ratio; ART-NP, Antiretroviral Naïve Patients; ART-EP, Antiretroviral Experience Patients.

**ART-Naive Patients**																			
	**2001 (12)**	**2002 (6)**	**2003 (20)**	**2004 (10)**	**2005 (17)**	**2006 (10)**	**2007 (25)**	**2008 (55)**	**2009 (47)**	**2010 (56)**	**2011 (52)**	**2012 (42)**	**2013 (60)**	**2014 (52)**	**2015 (62)**	**2016 (82)**	**2017 (82)**	**OR (95%CI)**	**P trend**
**PIs- Protease Inhibitors (n/%)**																			
**L90M**	6 (50.0)	1 (16.7)	5 (25.0)	3 (30.0)	2 (11.8)	0 (0.0%)	3 (12.0)	8 (14.5)	5 (10.6)	4 (7.1)	10 (19.2)	3 (7.1)	7 (11.7)	6 (11.5)	7 (11.3)	10 (12.2)	5 (6.1)	0.922 (0.875-0.971)	0.002
**M46IL**	1 (8.3)	1 (16.7)	3 (15.0)	1 (10.0)	3 (17.6)	1 (10.0)	1 (4.0)	4 (7.3)	3 (6.4)	8 (14.3)	5 (9.6)	2 (4.8)	4 (6.7)	5 (9.6)	1 (1.6%)	7 (8.5)	4 (4.9%)	0.942 (0.884-1.004)	0.067
**NNRTIs- Non-nucleoside reverse transcriptase inhibitors (n/%)**																			
**K103NS**	5 (41.7)	0 (0.0)	3 (15.0)	5 (50.0)	5 (29.4)	4 (40.0)	9 (36.0)	18 (32.7)	16 (34.0)	21 (37.5)	11 (21.2)	9 (21.4)	18 (30.0)	21 (40.4)	20 (32.3)	31 (37.8)	39 (47.6)	1.041 (1.002-1.083)	0.041
**G190ASEQ**	2 (16.7)	0 (0.0)	3 (15.0)	0 (0.0)	2 (11.8)	1 (10.0)	2 (8.0)	5 (9.1)	3 (6.4)	7 (12.5)	5 (9.6)	2 (4.8)	4 (6.7)	6 (11.5)	10 (16.1)	7 (8.5)	10 (12.2)	1.016 (0.956-1.080)	0.618
**K101EP**	2 (16.7)	0 (0.0)	1 (5.0)	0 (0.0)	1 (5.9)	1 (10.0)	2 (8.0)	6 (10.9)	5 (10.6)	6 (10.7)	2 (3.8)	2 (4.8)	1 (1.7)	2 (3.8)	5 (8.1)	5 (6.1)	4 (4.9)	0.955 (0.891-1.025)	0.202
**Y181CIV**	3 (25.0)	0 (0.0)	1 (5.0)	0 (0.0)	1 (5.9)	0 (0.0)	0 (0.0)	5 (9.1)	4 (8.5)	3 (5.4)	3 (5.8)	1 (2.4)	0 (0.0)	2 (3.8)	7 (11.3)	9 (11.0)	2 (2.4)	0.989 (0.917-1.066)	0.770
**NRTIs- Nucleoside reverse transcriptase inhibitors (n/%)**																			
**M184VI**	4 (33.3)	4 (66.7)	6 (30.0)	1 (10.0)	3 (17.6)	0 (0.0)	2 (8.0)	7 (12.7)	5 (10.6)	2 (3.6)	3 (5.8)	0 (0.0)	5 (8.3)	6 (11.5)	11 (17.7)	13 (15.9)	25 (30.5)	1.020 (0.967-1.075)	0.473
**M41L**	4 (33.3)	2 (33.3)	7 (35.0)	2 (20.0)	7 (41.2)	1 (10.0)	2 (8.0)	8 (14.5)	4 (8.5)	5 (8.9)	8 (15.4)	9 (21.4)	7 (11.7)	5 (9.6)	17 (27.4)	14 (17.1)	15 (18.3)	0.978 (0.933-1.025)	0.349
**D67NGE**	5 (41.7)	0 (0.0)	2 (10.0)	0 (0.0)	3 (17.6)	1 (10.0)	0 (0.0)	7 (12.7)	4 (8.5)	6 (10.7)	3 (5.8)	0 (0.0)	1 (1.7)	3 (5.8)	4 (6.5)	2 (2.4)	7 (8.5)	0.909 (0.850-0.971)	0.005
**T215 YFISCDVE**	5 (41.7)	1 (16.7)	3 (15.0)	1 (10.0)	5 (29.4)	0 (0.0)	0 (0.0)	2 (3.6)	1 (2.1)	0 (0.0)	1 (1.9)	0 (0.0)	1 (1.7)	2 (3.8)	6 (9.7)	3 (3.7)	11 (13.4)	0.939 (0.874-1.008)	0.084
**L210W**	2 (16.7)	1 (16.7)	3 (15.0)	2 (20.0)	1 (5.9)	0 (0.0)	0 (0.0)	0 (0.0)	0 (0.0)	1 (1.8)	1 (1.9)	2 (4.8)	4 (6.7)	4 (7.7)	3 (4.8)	5 (6.1)	5 (6.1)	0.983 (0.906-1.067)	0.687
**K219QERN**	2 (16.7)	0 (0.0)	0 (0.0)	0 (0.0)	4 (23.5)	1 (10.0)	1 (4.0)	5 (9.1)	4 (8.5)	5 (8.9)	4 (7.7)	1 (2.4)	2 (3.3)	3 (5.8)	6 (9.7)	5 (6.1)	10 (12.2)	1.005 (0.939-1.075)	0.895
**ART-Experienced Patients**																			
**PIs- Protease Inhibitors (n/%)**																			

	2001 (197)	2002 (303)	2003 (246)	2004 (332)	2005 (238)	2006 (278)	2007 (194)	2008 (134)	2009 (169)	2010 (129)	2011 (80)	2012 (62)	2013 (51)	2014 (78)	2015 (62)	2016 (55)	2017 (52)	OR (95%CI)	P trend
**M46IL**	46 (23.7)	66 (21.8)	40 16.3)	33 (9.9)	30 (12.6)	42 (15.1)	27 (13.9)	13 (9.7)	16 (9.5)	9 (7.0)	8 (10.0)	2 (3.2)	3 (5.9)	7 (9.0)	7 (11.3)	2 (3.6)	7 (13.5)	0.912 (0.884-0.941)	<0.001
**I54VLMTAS**	49 (25.3)	81 (26.7)	37 (15.0)	44 (13.3)	36 (15.1)	34 (12.2)	26 (13.4)	13 (9.7)	20 (11.8)	5 (3.9)	7 (8.8)	3 (4.8)	3 (5.9)	6 (7.7)	5 (8.1)	3 (5.5)	5 (9.6)	0.884 (0.856-0.913)	<0.001
**V82ATSF**	43 (22.2)	70 (23.1)	35 (14.2)	35 (10.5)	28 (11.8)	28 (10.1)	18 (9.3)	9 (6.7)	15 (8.9)	7 (5.4)	4 (5.0)	3 (4.8)	2 (3.9)	3 (3.8)	3 (4.8)	2 (3.6)	4 (7.7)	0.864 (0.833-0.897)	<0.001
**L90M**	69 (35.6)	97 (32.0)	49 (19.9)	52 (15.7)	41 (17.2)	37 (13.3)	26 (13.4)	13 (9.7)	10 (5.9)	6 (4.7)	8 (10.0)	4 (6.5)	6 (11.8)	5 (6.4)	5 (8.1)	3 (5.5)	4 (7.7)	0.844 (0.816-0.872)	<0.001
**NNRTIs- Non-nucleoside reverse transcriptase inhibitors (n/%)**																			
**K103NS**	53 (27.3)	83 (27.4)	80 (32.5)	131 (39.5)	111 (46.6)	119 (42.8)	94 (48.5)	52 (38.8)	74 (43.8)	46 (35.7)	24 (30.0)	25 (40.3)	20 (39.2)	25 (32.1)	23 (37.1)	18 (32.7)	24 (46.2)	1.023 (1.004-1.042)	0.020
**Y181CIV**	37 (19.1)	32 (10.6)	44 (17.9)	47 (14.2)	32 (13.4)	38 (13.7)	18 (9.3)	19 (14.2)	14 (8.3)	27 (20.9)	12 (15.0)	6 (9.7)	3 (5.9)	22 (28.2)	6 (9.7)	10 (18.2)	9 (17.3)	1.005 (0.979-1.031)	0.728
**G190ASE**	24 (12.4)	35 (11.6)	37 (15.0)	38 (11.4)	39 (16.4)	46 (16.5)	26 (13.4)	19 (14.2)	22 (13.0)	16 (12.4)	10 (12.5)	6 (9.7)	7 (13.7)	10 (12.8)	7 (11.3)	10 (18.2)	4 (7.7)	0.997 (0.970-1.024)	0.819
**K101PEH**	18 (9.3)	20 (6.6)	20 (8.1)	27 (8.1)	21 (8.8)	27 (9.7)	20 (10.3)	13 (9.7)	17 (10.1)	7 (5.4)	5 (6.3)	3 (4.8)	1 (2.0)	3 (3.8)	5 (8.1)	8 (14.5)	1 (1.9)	0.983 (0.949-1.017)	0.314
**NRTIs- Nucleoside reverse transcriptase inhibitors (n/%)**																			
**M41L**	76 (39.2)	119 (39.3)	80 (32.5)	85 (25.6)	64 (26.9)	62 (22.3)	38 (19.6)	18 (13.4)	27 (16.0)	6 (4.7)	8 (10.0)	8 (12.9)	4 (7.8)	5 (6.4)	5 (8.1)	6 (10.9)	6 (11.5)	0.850 (0.827-0.874)	<0.001
**D67NGE**	75 (38.7)	112 (37.0)	67 (27.2)	80 (24.1)	68 (28.6)	69 (24.8)	39 (20.1)	27 (20.1)	22 (13.0)	13 (10.1)	12 (15.0)	4 (6.5)	9 (17.6)	9 (11.5)	6 (9.7)	5 (9.1)	8 (15.4)	0.882 (0.860-0.905)	<0.001
**K70R**	39 (20.1)	54 (17.8)	32 (13.0)	49 (14.8)	44 (18.5)	43 (15.5)	24 (12.4)	16 (11.9)	9 (5.3)	5 (3.9)	9 811.3)	2 (3.2)	3 (5.9)	2 (2.6)	3 (4.8)	5 (9.1)	5 (9.6)	0.901 (0.873-0.931)	<0.001
**L210W**	37 (19.1)	50 (16.5)	41 (16.7)	29 (8.7)	23 (9.7)	21 (7.6)	18 (9.3)	7 (5.2)	8 (4.7)	1 (0.8)	1 (1.3)	1 (1.6)	1 (2.0)	1 (1.3)	1 (1.6)	2 (3.6)	1 (1.9)	0.813 (0.776-0.852)	<0.001
**T215YF**	90 (46.4)	139 (45.9)	93 (37.8)	96 (28.9)	73 (30.7)	57 (20.5)	41 (21.1)	22 (16.4)	23 (13.6)	9 (7.0)	11 (13.8)	5 (8.1)	4 (7.8)	6 (7.7)	4 (6.5)	3 (5.5)	8 (15.4)	0.824 (0.801-0.847)	<0.001
**K219QE**	58 (29.9)	76 (25.1)	57 (23.2)	55 (16.6)	59 (24.8)	52 (18.7)	40 (20.6)	22 (16.4)	15 (8.9)	10 (7.8)	10 (12.5)	5 (8.1)	4 (7.8)	11 (14.1)	12 (19.4)	13 (23.6)	9 (17.3)	0.935 (0.911-0.959)	<0.001
**M184IV**	129 (66.5)	185 (61.1)	159 (64.6)	220 (66.3)	160 (67.2)	199 (71.6)	136 (70.1)	93 (69.4)	107 (63.3)	78 (60.5)	47 (58.8)	38 (61.3)	34 (68.6)	51 (65.4)	40 (66.1)	39 (70.9)	29 (55.8)	0.995 (0.976-1.015)	0.639
**L74IV**	13 (6.7)	20 (6.6)	18 (7.3)	17 (5.1)	9 (3.8)	19 (6.8)	8 (4.1)	8 (6.0)	8 (4.7)	6 (4.7)	4 (5.0)	1 (1.6)	2 (3.9)	6 (7.7)	2 (3.2)	6 (10.9)	2 (3.8)	0.982 (0.942-1.023)	0.379

Table S4- Univariate analysis of factors associated with HIV transmitted drug resistance. TDR, transmitted drug resistance; NRTI, Nucleotide reverse transcriptase inhibitors; NNRTI, Non-nucleotide reverse transcriptase inhibitors; PI, Protease inhibitors; CI, confidence interval; OR, odds ratio.

	**Any TDR**			**NRTI TDR**			**NNRTI TDR**			**PI TDR**		
**Variable**	**n (%)**	**OR (95%CI)**	**p**	**n (%)**	**OR (95%CI)**	**p**	**n (%)**	**OR (95%CI)**	**p**	**n (%)**	**OR (95%CI)**	**p**
**Sex**												
Female *	215 (31.2%)			92 (31.8%)			135 (36.8%)			50 (24.3%)		
Male	467 (67.7%)	1.18 (0.99-1.40)	0.058	193 (66.8%)	1.13 (0.87-1.45)	0.359	228 (62.1%)	0.90 (0.72-1.12)	0.331	155 (75.2%)	1.68 (1.22-2.32)	0.002
**Age at diagnosis**												
18-25*	68 (9.9%)			22 (7.6%)			38 (10.4%)			19 (9.2%)		
26-40	291 (42.2%)	1.00 (0.76-1.32)	0.983	108 (37.4%)	1.15 (0.72-1.83)	0.559	151 (41.1%)	0.92 (0.64-1.33)	0.665	93 (45.1%)	1.14 (0.69-1.89)	0.596
41-55	223 (32.3%)	1.12 (0.84-1.49)	0.428	110 (38.1%)	1.73 (1.08-2.75)	0.021	114 (31.1%)	1.02 (0.70-1.48)	0.933	60 (29.1%)	1.07 (0.64-1.81)	0.798
>56	80 (11.6%)	1.08 (0.77-1.51)	0.671	29 (10.0%)	1.20 (0.69-2.12)	0.517	44 (12.0%)	1.06 (0.68-1.65)	0.814	29 (14.1%)	1.40 (0.78-2.52)	0.261
**Risk groups**												
Heterosexuals*	38 (5.5%)			17 (5.9%)			23 (6.3%)			11 (5.3%)		
MSM	18 (2.6%)	0.87 (0.48-1.56)	0.633	6 (2.1%)	0.64 (0.25-1.66)	0.362	6 (1.6%)	0.47 (0.19-1.17)	0.104	8 (3.9%)	1.36 (0.54-3.45)	0.512
IVDU	19 (2.8%)	0.94 (0.53-1.68)	0.838	9 (3.1%)	1.00 (0.44-2.30)	0.994	9 (2.5%)	0.73 (0.33-1.61)	0.432	6 (2.9%)	1.03 (0.38-2.84)	0.948
Others	5 (0.7%)	0.58 (0.22-1.52)	0.264	3 (1.0%)	0.80 (0.23-2.80)	0.730	1 (0.3%)	0.19 (0.02-1.42)	0.106	3 (1.5%)	1.26 (0.24-4.63)	0.726
**Geographic region of origin**												
Portugal*	249 (36.1%)			98 (33.9%)			121 (33.0%)			95 (46.1%)		
Europe	5 (0.7%)	0.60 (0.24-1.51)	0.280	4 (1.4%)	1.29 (0.46-3.60)	0.624	2 (0.5%)	0.50 (0.12-2.08)	0.344	0 (0.0%)	------	------
Sub-Saharan Africa	88 (12.8%)	0.91 (0.71-1.18)	0.489	45 (15.6%)	1.21 (0.84-1.73)	0.307	11 (3.0%)	1.31 (0.95-1.80)	0.094	10 (4.9%)	0.27 (0.14-0.51)	<0.001

	Any TDR			NRTI TDR			NNRTI TDR			PI TDR		
**Variable**	**n (%)**	**OR (95%CI)**	**p**	**n (%)**	**OR (95%CI)**	**p**	**n (%)**	**OR (95%CI)**	**p**	**n (%)**	**OR (95%CI)**	**p**
South America	20 (2.9%)	1.01 (0.63-1.64)	0.953	6 (2.1)	0.76 (0.33-1.77)	0.531	11 (3.0%)	1.16 (0.61-2.18)	0.656	1 (0.5%)	0.52 (0.19-1.43)	0.207
**Subtypes**												
B*	304 (44.1%)			141 (48.8%)			129 (35.1%)			105 (51.0%)		
Non-B	240 (34.8%)	0.70 (0.59-0.84)	<0.001	78 (27.0%)	0.50 (0.37-0.66)	<0.001	164 (44.7%)	1.18 (0.93-1.49)	0.173	62 (30.1%)	0.53 (0.389-0.73)	<0.001
**CD4** (cells/mL)												
<50*	36 (5.2%)			18 (6.2%)			20 (5.4%)			10 (4.9%)		
51-200	63 (9.1%)	0.95 (0.62-1.46)	0.810	23 (8.0%)	0.69 (0.37-1.29)	0.241	40 (10.9%)	1.09 (0.63-1.90)	0.749	15 (7.3%)	0.81 (0.36-1.83)	0.617
201-350	72 (10.4%)	1.03 (0.68-1.57)	0.885	29 (10.0%)	0.82 (0.45-1.50)	0.523	39 (10.6%)	1.00 (0.58-1.74)	0.992	22 (10.7%)	1.14 (0.53-2.42)	0.743
351-500	75 (10.9%)	1.20 (0.79-1.81)	0.399	23 (8.0%)	0.71 (0.38-1.34)	0.291	45 (12.3%)	1.29 (0.75-2.21)	0.357	27 (13.1%)	1.54 (0.74-3.22)	0.247
>501	100 (14.5%)	1.27 (0.85-1.89)	0.241	39 (13.5%)	0.97 (0.55-1.71)	0.907	49 (13.4%)	1.10 (0.64-1.88)	0.727	33 (16.0%)	1.49 (0.73-3.06)	0.247
**Viral Load** (log_{10} copies/ml)												
< 4.0*	128 (18.6%)			65 (22.5%)			76 (20.7%)			41 (19.9%)		
4.1 to 5.0	216 (31.3%)	0.78 (0.62-0.98)	0.032	80 (27.7%)	0.57 (0.40-0.79)	0.001	112 (30.5%)	0.68 (0.50-0.92)	0.012	71 (34.5%)	0.81 (0.55-1.20)	0.296
>5.1	176 (25.5%)	0.67 (0.53-0.86)	0.001	64 (22.1%)	0.48 (0.34-0.69)	<0.001	91 (24.8%)	0.59 (0.43-0.81)	0.001	46 (22.3%)	0.56 (0.37-0.86)	0.008

* **Reference category**

Table S5- Association for the most prevalent drug-resistance mutation for each major drug class: M184V, L90M, and K103N for NRTI, PI, and NNRTI with viral load for ART-NP. CI, confidence interval; OR, odds ratio; ART-NP, Antiretroviral Naïve Patients

	**M184V**		**K103NS**		**L90M**	
**Variable**	**OR (95%CI)**	**p**	**OR (95%CI)**	**p**	**OR (95%CI)**	**p**
**Viral Load** (log_{10} copies/ml)						
< 4.0*						
4.1 to 5.0	0.30 (0.17-0.54)	<0.001	0.76 (0.51-1.12)	0.164	0.64 (0.35-1.16)	0.145
>5.1	0.15 (0.07-0.33)	<0.001	0.62 (0.41-0.94)	0.023	0.40 (0.20-0.78)	0.008

[*] **Reference category**

Table S6- Univariate analysis of factors associated with HIV acquired drug resistance. ADR, acquired drug resistance; NRTI, Nucleotide reverse transcriptase inhibitors; NNRTI, Non-nucleotide reverse transcriptase inhibitors; PI, Protease inhibitors; CI, confidence interval; OR, odds ratio.

	Any ADR			NRTI ADR			NNRTI ADR			PI ADR		
**Variable**	**n (%)**	**OR (95%CI)**	**p**	**n (%)**	**OR (95%CI)**	**p**	**n (%)**	**OR (95%CI)**	**p**	**n (%)**	**OR (95%CI)**	**p**
**Sex**												
Female *	787 (29.7%)			641 (22.8%)			538 (30.5%)			208 (22.9%)		
Male	1861 (70.0%)	1.45 (1.27-1.70)	<0.001	1575 (70.8%)	1.45 (1.26-1.66)	<0.001	1217 (69.0%)	1.16 (1.01-1.33)	0.036	699 (76.9%)	1.83 (1.54-2.18)	<0.001
**Age at diagnosis**												
18-25*	89 (3.3%)			18 (2.0%)			67 (3.8%)			18 (2.0%)		
26-40	1336 (50.3%)	1.62 (1.15-2-26)	0.005	443 (48.7%)	1.44 (1.04-2.00)	0.030	886 (50.3%)	1.09 (0.78-1.52)	0.613	443 (48.7%)	2.23 (1.35-3.70)	0.002
41-55	937 (35.3%)	1.57 (1.12-2.21)	0.009	332 (36.5%)	1.45 (1.04-2.03)	0.029	621 (35.2%)	1.07 (0.76-1.50)	0.694	332 (36.5%)	2.41 (1.45-4.00)	0.001
>56	252 (9.5%)	2.06 (1.38-3.08)	<0.001	103 (11.3%)	1.93 (1.31-2.84)	0.001	160 (9.1%)	1.14 (0.78-1.68)	0.501	103 (11.3%)	3.26 (1.89-5.61)	<0.001
**Risk groups**												
Heterosexuals*	139 (5.2%)			74 (3.3%)			83 (4.7%)			56 (6.2%)		
MSM	42 (1.6%)	0.47 (0.26-0.85)	0.013	1108 (49.8%)	0.38 (0.22-0.67)	0.001	27 (1.5%)	0.76 (0.43-1.33)	0.339	13 (1.4%)	0.52 (0.26-1.02)	0.058
IVDU	139 (5.2%)	1.24 (0.74-2.06)	0.416	787 (35.4%)	1.15 (0.74-1.81)	0.532	96 (5.4%)	1.47 (0.97-2.24)	0.070	48 (5.3%)	0.86 (0.54-1.36)	0.510
Others	78 (2.9%)	0.84 (0.48-1.46)	0.541	219 (9.8%)	0.85 (0.52-1.41)	0.531	51 (2.9%)	1.10 (0.68-1.77)	0.712	32 (3.5%)	0.96 (0.57-1.62)	0.894
**Geographic region of origin**												
Portugal*	711 (26.8%)			564 (25.3%)			472 (26.8%)			244 (26.8%)		
Europe	5 (0.2%)	0.21 (0.08-0.57)	0.002	4 (0.2%)	0.26 (0.09-0.78)	0.017	4 (0.2%)	0.36 (0.12-1.08)	0.069	1 (0.1%)	0.20 (0.03-1.47)	0.113

	Any ADR			NRTI ADR			NNRTI ADR			PI ADR		
**Variable**	**n (%)**	**OR (95%CI)**	**p**	**n (%)**	**OR (95%CI)**	**p**	**n (%)**	**OR (95%CI)**	**p**	**n (%)**	**OR (95%CI)**	**p**
Sub-Saharan Africa	263 (9.9%)	1.00 (0.79-1.25)	0.963	212 (9.5%)	1.03 (0.82-1.28)	0.807	191 (10.8%)	1.17 (0.93-1.46)	0.179	59 (6.5%)	0.60 (0.44-0.81)	0.001
South America	30 (1.1%)	1.16 (0.63-2.16)	0.632	25 (1.1)	1.24 (0.69-2.24)	0.474	20 (1.1%)	1.11 (0.61-2.01)	0.735	6 (0.7)	0.56 (0.23-1.33)	0.189
**Subtypes**												
B*	1207 (45.4%)			1055 (47.4%)			767 (43.5%)			473 (52.0%)		
Non-B	1020 (38.4%)	0.70 (0.60-0.82)	<0.001	819 (36.8%)	0.63 (0.55-0.73)	<0.001	715 (40.6%)	0.99 (0.86-1.14)	0.862	316 (34.8%)	0.64 (0.54-0.75)	<0.001
**CD4** (cells/mL)												
<50*	214 (8.1%)			166 (7.5%)			162 (9.2%)			64 (7.0%)		
51-200	572 (21.5%)	0.85 (0.64-1.12)	0.252	471 (21.2%)	1.02 (0.78-1.32)	0.912	399 (22.6%)	0.77 (0.59-1.00)	0.046	153 (16.8%)	0.82 (0.59-1.13)	0.221
201-350	545 (20.5%)	0.88 (0.66-1.17)	0.376	448 (20.1%)	1.04 (0.80-1.35)	0.778	358 (20.3%)	0.70 (0.54-0.91)	0.009	192 (21.1%)	1.17 (0.85-1.61)	0.332
351-500	392 (14.8%)	1.22 (0.90-1.66)	0.206	328 (14.7%)	1.37 (1.03-1.82)	0.030	259 (14.7%)	0.85 (0.64-1.13)	0.263	139 (15.3%)	1.34 (0.96-1.88)	0.084
>501	402 (15.1%)	1.08 (0.80-1.47)	0.605	356 (16.0%)	1.46 (1.10-1.93)	0.009	252 (14.3%)	0.73 (0.55-0.96)	0.024	156 (17.2%)	1.45 (1.04-2.02)	0.027
**Viral Load** (log_{10} copies/ml)												
< 4.0*	1164 (43.8%)			1023(46.0%)			742 (42.1%)			410 (45.1%)		
4.1 to 5.0	850 (32.0%)	0.70 (0.59-0.83)	<0.001	706 (31.7%)	0.67 (0.57-0.78)	<0.001	573 (32.5%)	0.93 (0.80-1.08)	0.315	302 (33.2%)	0.89(0.75-1.06)	0.179
>5.1	346 (13.0%)	0.39 (0.32-0.48)	<0.001	247 (11.1%)	0.33 (0.27-0.40)	<0.001	257 (14.6%)	0.75 (0.62-0.90)	0.003	96 (10.6%)	0.50 (0.39-0.64)	<0.001

* **Reference category**
